# Supplementary material for: Validation of CIP2A as a Biomarker of Subsequent Disease Progression and Treatment Failure in Chronic Myeloid Leukaemia
Source: Cancers (Basel). 2021 Apr 29;13(9):2155. doi: 10.3390/cancers13092155 (PMC8124525; doi:10.3390/cancers13092155)
Supplement: Supplementary file 1 [file cancers-13-02155-s001.zip › cancers-1146267-supplementary.pdf]

cancers

Supplementary Materials: Validation of CIP2A as a Biomarker of Subsequent Disease Progression and Treatment Failure in Chronic Myeloid Leukaemia

Richard E. Clark, Ammar A. Basabrain, Gemma M. Austin, Alison K. Holcroft, Sandra Loaiza, Jane F. Apperley, Christopher Law, Laura Scott, Alexandra D. Parry, Laura Bonnett and Claire M. Lucas

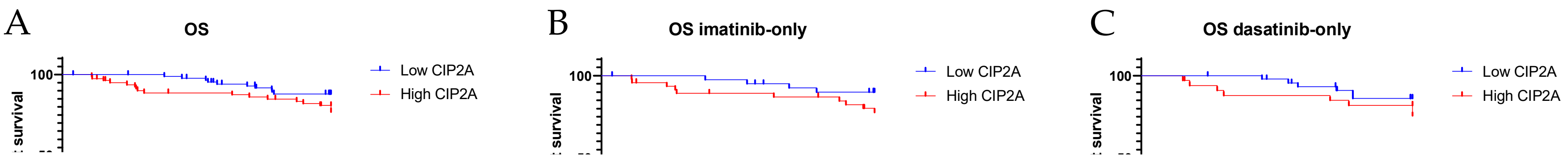

Figure S1. Overall survival. Kaplan-Meier curves for overall survival stratified by diagnostic CIP2A level for (**A**) all 172 patients, (**B**) imatinib recipients only, and (**C**) dasatinib recipients only. *p* values were determined using the Log-rank (Mantel-Cox) test; *p* values are shown where significant.
